# Digestive activity and organic compounds of *Nezara viridula* watery saliva induce defensive soybean seed responses

**DOI:** 10.1038/s41598-020-72540-3

**Published:** 2020-09-22

**Authors:** Romina Giacometti, Vanesa Jacobi, Florencia Kronberg, Charalampos Panagos, Arthur S. Edison, Jorge A. Zavala

**Affiliations:** 1 Consejo Nacional de Investigaciones Científicas y Técnicas / Instituto de Investigaciones en Biociencias Agrícolas y Ambientales, Facultad de Agronomía, Universidad de Buenos Aires, Avda. San Martín 4453, C1417DSE Buenos Aires, Argentina; 2 Cátedra de Bioquímica, Facultad de Agronomía, Universidad de Buenos Aires, Avda. San Martín 4453, C1417DSE Buenos Aires, Argentina; 3 Complex Carbohydrate Research Center (CCRC), University of Georgia, Athens, GA USA

**Keywords:** Chemical biology, Plant sciences, Zoology

## Abstract

The stink bug *Nezara viridula* is one of the most threatening pests for agriculture in North and South America, and its oral secretion may be responsible for the damage it causes in soybean (*Glycine max*) crop. The high level of injury to seeds caused by pentatomids is related to their feeding behavior, morphology of mouth parts, and saliva, though information on the specific composition of the oral secretion is scarce. Field studies were conducted to evaluate the biochemical damage produced by herbivory to developing soybean seeds. We measured metabolites and proteins to profile the insect saliva in order to understand the dynamics of soybean-herbivore interactions. We describe the mouth parts of *N. viridula* and the presence of metabolites, proteins and active enzymes in the watery saliva that could be involved in seed cell wall modification, thus triggering plant defenses against herbivory. We did not detect proteins from bacteria, yeasts, or soybean in the oral secretion after feeding. These results suggest that the digestive activity and organic compounds of watery saliva may elicit a plant self-protection response. This study adds to our understanding of stink bug saliva plasticity and its role in the struggle against soybean defenses.

## Introduction

The southern green stink bug *Nezara viridula* L. is an important pest, since it invades worldwide soybean (*Glycine max*) crops, with the south of the U.S. and South America being the main focus of infestation^1,2^. In order to protect crops from insects, technologies like BT (*Bacillus thuringiensis*) transgenic plants^3^, or dsRNA (double-stranded RNA) for gene silencing are being exploited^4^. However, worldwide primary management strategy to limit stink bug population still relies on the application of insecticides, an unfriendly environmental agronomical practice, which is also not very efficient due to the development of resistance in insects^5,6^. Due to their ability to adapt to continuous changing environments and their polyphagous activity, stink bugs have become cosmopolitan insects. Although most species have a wide host range, soybean is often the preferred host^7^. Stink bug adults live and develop within the plant, feeding from developing soybean seeds. When the season changes, stink bugs shift their preference from the primary host to a wide variety of plant species^8^.

Piercing-sucking insects like *N. viridula* lacerate and inject toxic saliva on developing seeds, causing cotyledon damage and massive economic losses due to yield reduction^9^. The insects’ oral secretion is one of the first fluids to come in contact with the plant during herbivory and has been thoroughly studied in caterpillars^10^ and aphids^11^. Though some information on gut and salivary glands of piercing-sucking insects is available^12–15^, the composition of *Nezara* saliva has not been studied. Most research about digestive enzyme secretion in insects has been conducted on aphid midgut cells^16^ or oral secretions and regurgitant of caterpillars. This has led to the identification of proteins like β-glucosidase^17,18^, peptides named inceptins^19,20^, and the well-known amino acid-fatty acid conjugates (FACs), while less is known about effectors^20^.

Stink bugs use their stylets to penetrate the developing seeds and draw nutrients, and effectors may be injected directly into the tissue along with the saliva. As previously described in the stink bug *Halyomorpha halys*, jelling saliva contains both insect- and plant-derived proteins. This jelling secretion forms the sheath, which quickly sets and seals the puncture site^12^. It has also been reported that *N. viridula’s* gut cells expresses high cysteine protease levels, while salivary glands express high levels of serine proteases and nucleases^14^. Since these stink bug enzymes can be inhibited by soybean protease inhibitors accumulated in seeds, *N. viridula* prefer to feed on developing seeds without induced defenses^21^.

Whereas the impact of watery saliva of chewing herbivores on plant responses is well studied, much less is known about the effects of saliva effectors from piercing-sucking insects, such as stink bugs. Enzymes in the saliva of *N. viridula* may be responsible for inducing indirect defenses in soybean, similar to the production of volatiles like sulcatol and sulcatone in wheat damaged plants by aphid salivary pectinase^22^. It was also shown that polyphenol oxidase in aphid saliva triggered jasmonic acid (JA)-related defense responses in wheat^23^. Although plant defense reactions to piercing-sucking insects may be quite different, they frequently comprise salicylic acid (SA)-signaling mediated response. However, it was reported in *H. halys* that the oral secretions did not affect the expression of pathogenesis related genes, which are inducible by salicylates^12^. We have previously shown that developing soybean seeds respond to *N. viridula’s* attack by recognizing the saliva and triggering JA/ethylene (ET)- and SA-regulated defenses through the mitogen activated protein kinases (MAPK) pathway^21^. After saliva detection, soybean seeds induced tailored defenses, resulting in a decreased preference for previously attacked seeds^21,24^. However, watery saliva effectors and their effects on defensive responses of plants after stink bug feeding are still not well known. Characterizing active compounds of saliva will help to elucidate possible effectors that induce plant responses.

To study the dynamics between stink bugs and developing seeds upon soybean herbivory we describe here the morphology of the mouthparts of *N. viridula* and their impact on soybean cotyledons. In addition, we employed nuclear magnetic resonance (NMR) spectroscopy to profile *N. viridula’s* watery saliva, which allowed us to identify the presence of certain metabolites, such as organic acids and amino acids. Mass spectrometry (MS) proteomics showed that a large percentage of the proteins identified in the watery saliva function were digestive enzymes, proteins involved in signal transduction, nucleotide binding and oxidoreductase activity, among other categories. Our results suggest that the watery saliva of *Nezara* is more like an enzymatic secretion rather than a liquid with inactive compounds, and some of the metabolites and proteins in it could be eliciting specific soybean mediated defenses against the southern green stink bug.

## Results

### *Nezara viridula* physiology and feeding detrimental effects on soybean seeds

**Figure 1:**
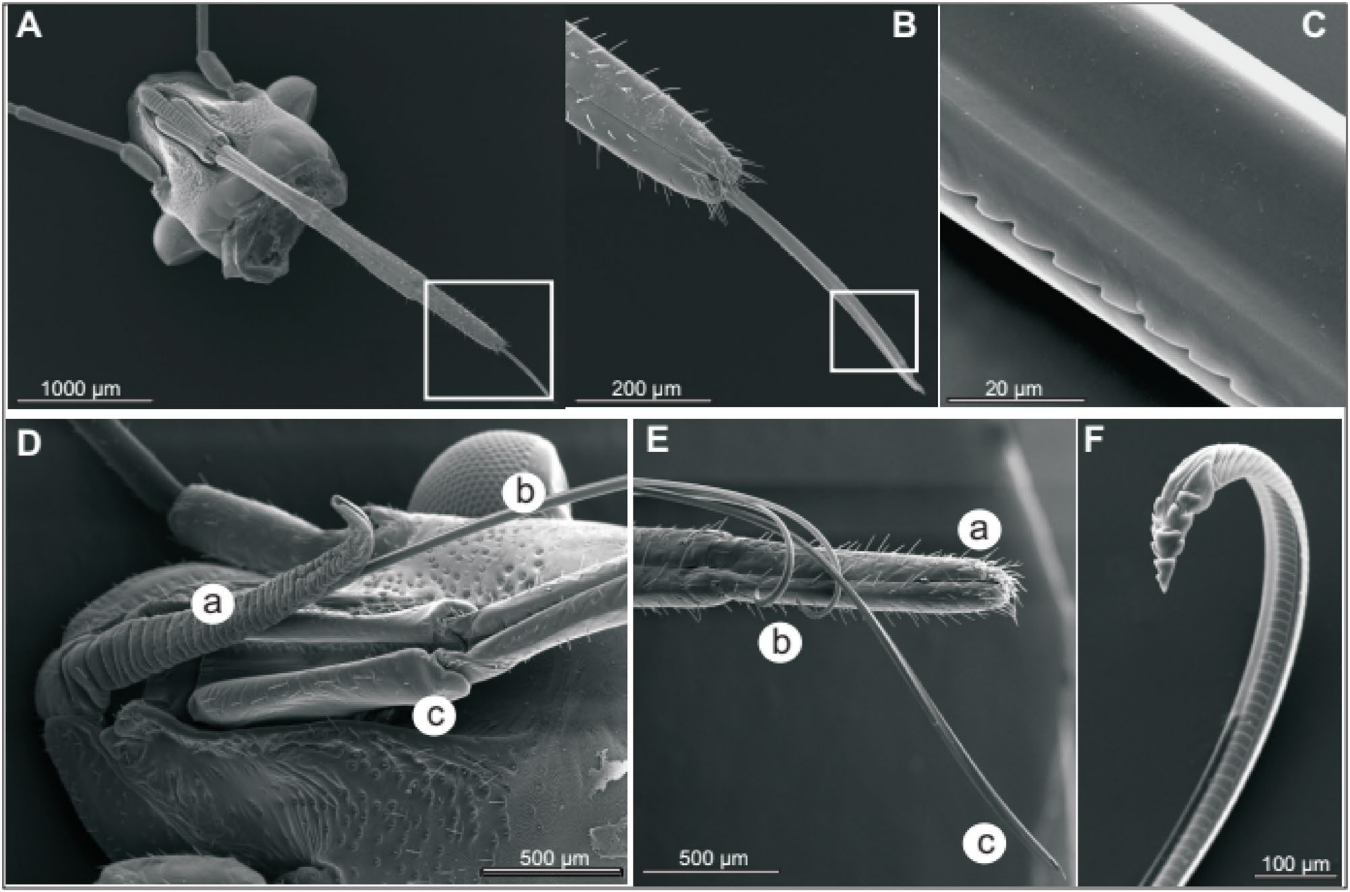
Scanning electron microscopy of mouthparts of an adult specimen of *Nezara viridula*. (**A**) anterior view of the head of the stink bug; (**B**) beak and mandible tip; (**C**) enlarged view of the mandible tip; (**D**) lateral view of the head with details of: (a) central labrum, (b) stylets, (c) first labial segment; (**E**) detailed stylet fascicle showing: (a) last segment of the beak, (b) mandibles, (c) maxillary stylets; (**F**) detail of teeth in one mandibular stylet.

To enhance our understanding of the physiology and feeding behaviour of the green stink bug and to provide additional information on the ecological impact of the interaction between *N. viridula* and the developing soybean pods and seeds, we described the morphology of the mouthparts of this stink bug involved in probing and feeding. Green stink bug mouthparts observed by Scanning Electron Microscopy (SEM) are sophisticated and resemble those described in other piercing-sucking insects, composed of the labium, labrum and a stylet fascicle housed by a long beak (Fig. 1A). The stylet presents a set of two separated inner maxillaries and two outer mandibular serrate-edged stylets (Fig. 1B–F), keeping the food canal separated from the lateral salivary canals inside of the mandibles (Fig. 1F).

**Figure 2:**
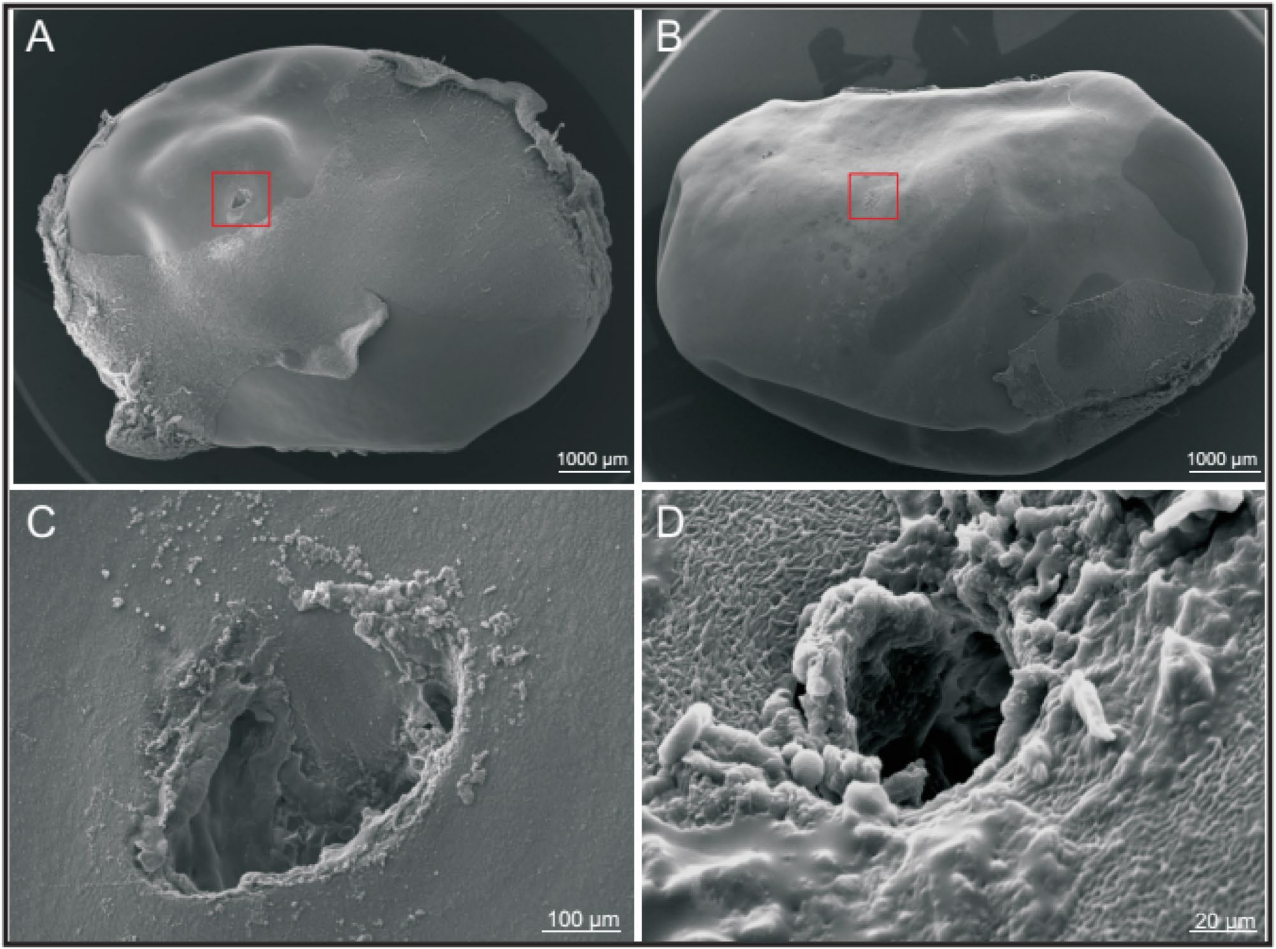
Damage produced in soybean developing seed’s structure by herbivory. SEM comparison of the damage produced in the developing fresh tissue by: (**A**) the puncture of a needle, mimicking *Nezara’s* stylet, and (**B**) the damage produced by the insect’s feeding activity; (**C**,**D**) offer a detailed view of the wounded area by mechanical damage and the insect, respectively.

In fresh developing soybean seeds, we used SEM to compare the effect of two different types of wounds, one produced by the insect feeding and another by mechanical damage made with a needle to mimic the stink bug’s stylet but in absence of the oral secretions (Fig. 2). At lower magnification, the outer structure of the cotyledon mechanically damaged by the needle appears to collapse around the site of insertion, revealing sharp round clear edges (Fig. 2A,C), while after herbivory treatment the seed’s surface showed a relatively smooth appearance (Fig. 2B). Zooming into the site where the stylet made the damage, a different texture surrounding the cavity was observed in comparison to the seed damaged with the needle (Fig. 2D).

**Figure 3:**
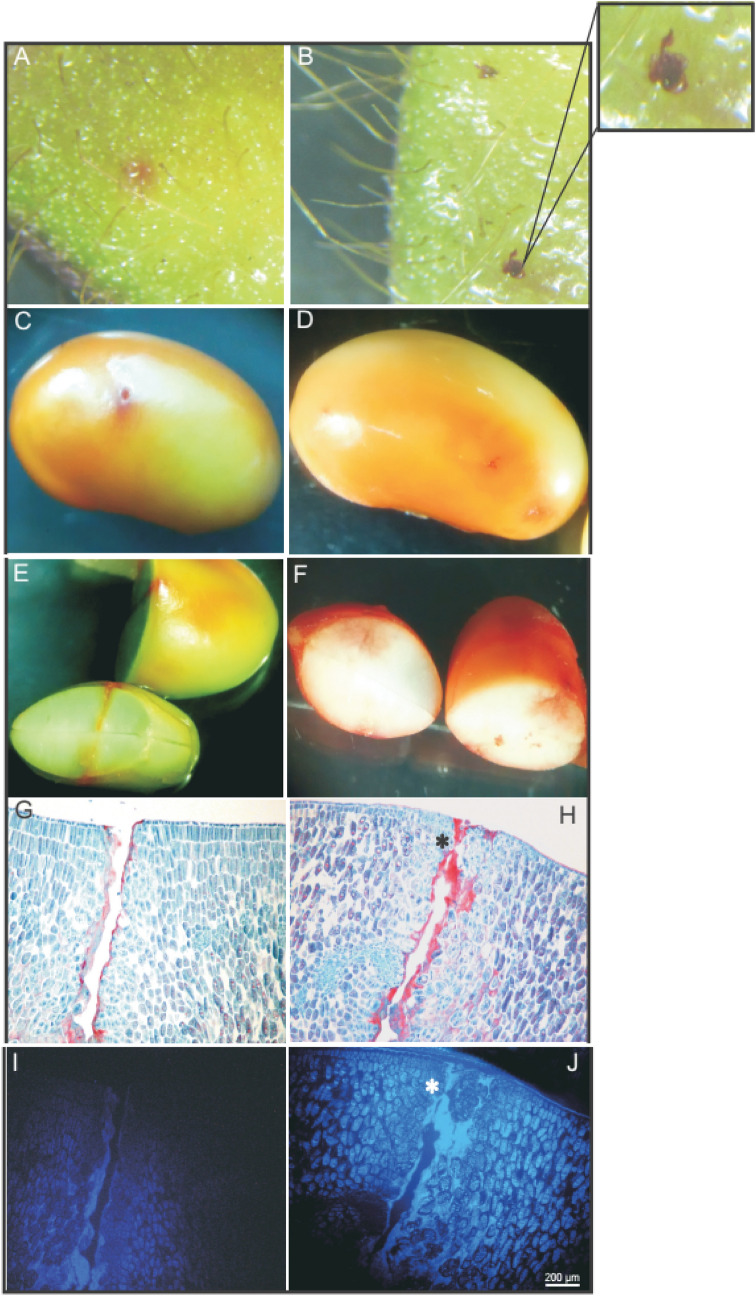
Soybean tissue response to *Nezara viridula* feeding activity. Acid fuchsine staining of damaged pods: (**A**) mechanical damage, and (**B**) attacked by the stink bug, an inset of the pod surface is shown to point the jellified saliva sheath left on the surface. Soybean stained cotyledons showing in (**A**,**E**) mechanical damage, (**D**,**F**) damage produced after insect injecting saliva. Light photomicrographs and histological analysis of cross sections of (**G**) mechanical damaged seed and (**H**) attacked by the insect. Same sections stained with safranin showing (**I**) auto-fluorescence and (**J**) bright lignified cells in the puncture site and a siege marked with an asterisk.

*Nezara viridula* produced two different types of oral secretions, a jellified one that helps to form a salivary sheath sealing the puncture site, and the watery or enzymatic saliva that the insect injects in order to dissolve the tissues (Fig. 3). Staining the treated pods and seeds with an acid fuchsin solution enabled us to observe both the injury and the presence of the salivary sheaths left only on the pods after stink bug feeding (Fig. 3A,B). Inspection of stained seeds allowed the detection of feeding damage versus mechanical damage (Fig. 3C,D). In contrast to the clean penetrating wound produced by the needle, the feeding activity resulted in widespread damage (Fig. 3E,F).

Histological analysis of the seeds by safranin-fast green-staining indicated damage at the sharp entrance of the needle, although this damage was contained in the first line of cells. Herbivory, on the other hand, led to both the destruction of cells caused by the drilling of the stylets into the cotyledon and dissolution by the injected oral secretion of the tissue content even further away from the boring area (Fig. 3G,H). Under UV-light in a fluorescence microscope the same safranin-stained sections revealed brighter autofluorescence for developing seeds after herbivory treatment in comparison with seeds subjected to mechanical damage (Fig. 3I,J), showing an increase in cell wall thickness in the lacerated area. Furthermore, mature seeds exposed to the stink bug were harvested and analysed by SEM (SI Fig. 1). Results revealed the extent of the damage that the insect caused underneath the tegument in the seed architecture, affecting the antioxidant balance of the tissue, and therefore the viability and germinative power of the seeds (SI Fig. 1).

### Metabolite composition of *N. viridula* watery saliva

**Figure 4:**
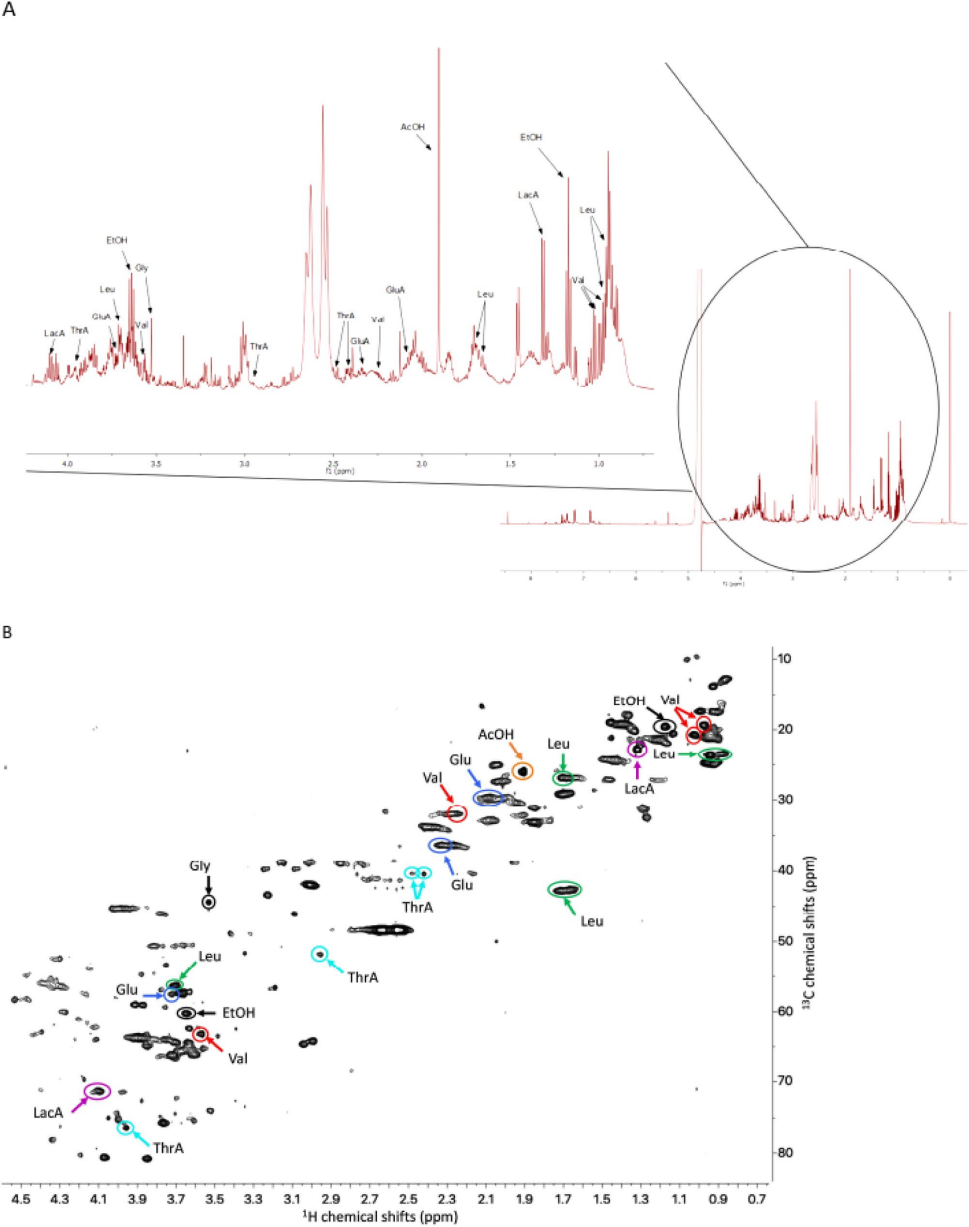
NMR analysis of *N. viridula* saliva. Stink bug’s watery oral secretion from a group of 200 adults was collected under laboratory conditions and subjected to NMR analysis. (**A**) 600 MHz proton spectra of watery saliva. An enlarged view of signals at 4 and 1 ppm is shown. (**B**) Representative 2-dimensional ^1^H–^13^C HSQC NMR spectrum of oral secretion. Complete ^1^H and ^13^C chemical shift assignments are listed in Table 1.

The different responses of the seeds subjected to either herbivory or mechanical damage found in this study led us to analyse the chemical composition of *N. viridula* saliva. The NMR spectra of the oral secretion of the stink bug showed relatively good resolution for the abundant metabolites. The combination of the 2D ^13^C-HSQC (Heteronuclear Single-Quantum Correlation) and ^13^C-HSQC-TOCSY (Heteronuclear Single-Quantum Correlation-Total Correlation Spectroscopy) spectra (Fig. 4) were used to assign metabolites in the spectrum (Table 1). Despite very limited amounts of saliva from just 200 stinkbugs, we were able to detect and approximately quantify several primary metabolites that ranged in concentration from about 30 to 500 µM (Table 1; Fig. 4). These included amino acids (glutamate, leucine, glycine, and valine), organic acids (lactic, acetic, and threo-isocitric acids), and ethanol.

**Table 1:** List of metabolites found in *Nezara viridula* oral secretion.

Metabolite	Confidence level^a^	Chemical shifts (^1^H ppm/^13^C ppm)	Concentration (mM)
Glutamic acid	4	3.72/57.48, 2.34/36.36, 2.09/29.68	0.04
Threo-isocitric acid	4	3.95/76.47, 2.95/51.85, 2.42–2.48/40.42	0.03
Leucine	4	3.70/56.35, 1.69/42.78, 1.70/26.89, 0.95/23.65	0.15
Lactic acid	4	4.10/71.30, 1.32/22.84	0.06
Ethanol	4	3.64/60.22, 1.17/19.61	0.08
Glycine	3	3.53/44.43	0.29
Acetic acid	3	1.91/26.09	0.11
Valine	3	3.56/63.28, 2.25/32.04, 1.03/20.78, 0.97/19.33	0.48

^a^Confidence level of the annotation is given using the following scale: Level 1: Matching with literature Chemical Shift values, Level 2: ^1^H 1D Chemical Shift matching, Level 3: ^1^H^13^C 2D HSQC Chemical Shift matching, Level 4: ^1^H^13^C 2D HSQC-TOCSY matching, and Level 5: spiking with an authentic standard (not done in this study).

### Analysis of *N. viridula* oral secretion by proteomic analysis

**Figure 5:**
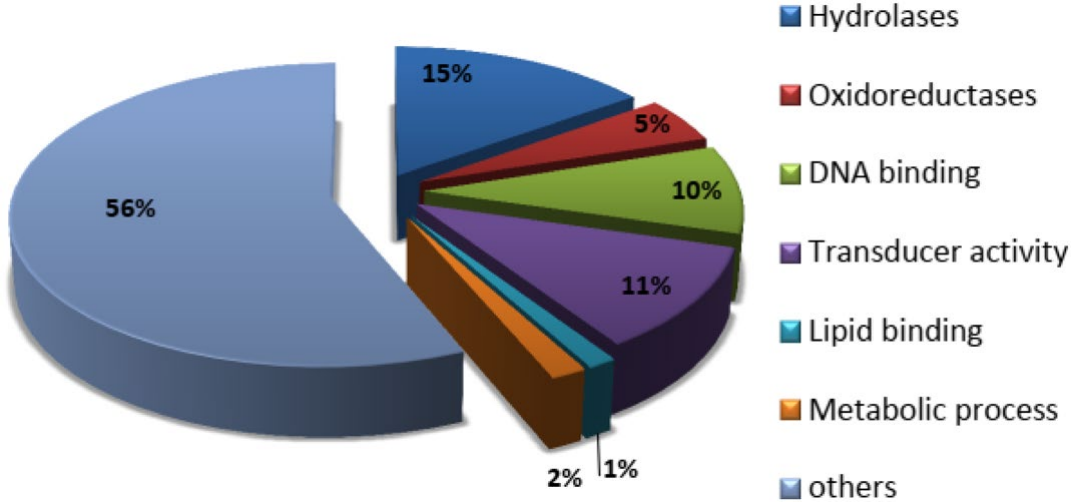
Proteomic identification of the components in the stink bugs saliva. To identify proteins in the watery saliva, samples were digested and Mass Spectrometry (MS) analyses were performed. Functional annotation and relative abundance of enzymes identified from stinks bug saliva proteome are shown. Only high confidence peptide matches with a maximum protein and peptide false discovery rate of 1% were selected through a reverse database approach.

For valid protein identification in the watery saliva, we based the analysis on the presence of the same peptides in all the independent biological replicates evaluated by LC-ESI/MS (Liquid Chromatography-Electrospray Mass Spectrometry, Orbitrap). Following these criteria, our proteomic data showed that 15% of the stink bug’s oral secretion is composed of digestive enzymes (Fig. 5). Also 11% of the proteins detected were related to signal transduction pathways, and 10% of the total proteins contained conserved domains for ATP and nucleotide binding. In addition, 5% were enzymes involved in antioxidant processes, including the presence of some peroxidases, while 2% corresponded to peptides associated with metabolic functions, and 1% of the hits were linked to lipid binding proteins (Fig. 5). In Table 2 we identified nine hydrolases, several of them amylases, a trehalase and peptidases, most of them carrying signal peptides. Also, six more enzymes involved in catalysing oxide-reduction processes, like malate dehydrogenase, peroxidase and cytochrome oxidases were identified (Table 2). Furthermore, when peptides were searched against soybean, bacteria or yeast databases, there was no sign of the presence of proteins coming from any of these organisms (data not shown).

**Table 2:** Most relevant enzymes identified in the saliva of the green stink bug by nano LC-ESI/MS analysis.

UniProt Accesion no	Protein biological function (GO)	Insect species	MW (KDa)	Sequence coverage
**Hydrolases**				
O18345	Alpha-amylase 2	*Drosophila ananassae*	53.5	4.5
A0A1I8P9Y3	Metallo endopeptidase	*Stomoxys calcitrans*	80	3.2
A0A0A1X8Q6	Alpha-amylase	*Zeugodacus cucurbitae*	65.1	4.2
Q16V81	Trehalase	*Aedes aegypti*	70.8	1.6
A0A1L8EBI2	Putative alpha-amylase	*Haematobia irritans*	68	5.5
Q23937	Amylase	*Drosophila ficusphila*	16.2	14.3
A0A0C9QMF1	Metallopeptidase	*Fopiusarisanus*	72.7	3.9
Q8I057	Amylase	*Drosophila birchii*	38.8	3.7
A0A1Q1NPH6	Serine-type endopeptidase	*Pristhesancus plagipennis*	34.2	2.6
**Oxidoreductases**				
A0A159VDU8	Cytochrome c oxidase	*Xylophagus sp*	20.9	20.4
J9XJL5	Malate dehydrogenase	*Cossedia hyriodes*	14.5	32.6
G1FCE5	Cytochrome P450	*Bemisia tabaci*	56.9	7.5
A0A182RFW9	Cytochrome P450	*Anopheles funestus*	57,7	5.7
A0A182GJY5	Oxidoreductase	*Aedes albopictus*	36,3	7.3
A0A1B6MIA0	Peroxidase	*Graphocephala atropunctata*	77.4	1.6

**Figure 6:**
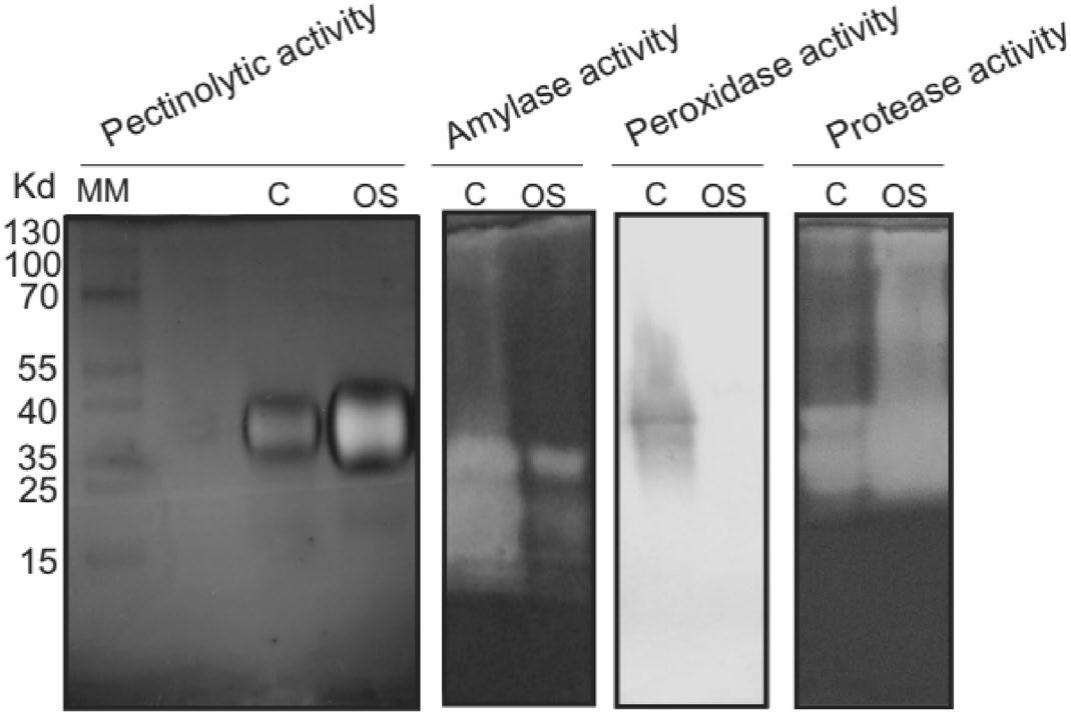
Zymogram analysis of several enzymes expressed in *Nezara´s* oral secretion. SDS-PAGE gel with 1% pectin co-polymerized as a substrate was used to test for pectinolytic activity, OS, corresponds to the oral secretion sample and C, to the enzyme pectinase from *Aspergillus niger* (Sigma) used as a positive control. For amylolytic activity the gel was co-polymerized with 0.5% starch, α-amylase from *Aspergillus oryzae* (Sigma) was included as positive control. For peroxidase activity a native gel was used and 10 µg of protein from soybean seeds extract was included as positive control. Protease activity was detected in a gelatin co-polymerized native gel, and bovine trypsin (Sigma) was included as positive control. A pre-stained molecular weight marker was used (MM, Kaleidoscope, Bio-Rad). One of three independent experiments is shown for each enzyme tested.

To further authenticate the proteomic results, zymogram assays were performed on the extracted saliva of *N. viridula* to give insight into the enzymes being expressed and active in the fluid (Fig. 6). While saliva was positive for pectinase, amylase and proteases activities, we could not detect peroxidase activity under native PAGE conditions (Fig. 6), which was observed in the proteomic analyses detailed above (Table 2).

## Discussion

Stink bugs use their oral secretion as an efficient way to digest and absorb the nutritional value from plant tissues and seeds content, but also to inhibit plant defenses^25^. Despite the detrimental impact that insect feeding has on agriculture, little research has been reported on the oral secretions of stink bugs. Most previous studies focused on combinations of oral secretions and regurgitates from chewing insects like caterpillars^19,20,26–28^. Research on piercing-sucking insects—with the exception of a study on the brown marmorated stink bug (*Halyomorpha halys*)^13^—has mostly focused on intact salivary gland tissue rather than the isolated saliva^12,15^. Thus, it is possible that in previous studies, the identified compounds and cellular proteins were from both accessory and principal glands.

In this study we described the mouthparts of *N. viridula* that inflicted damage and injected watery saliva into soybean developing seeds. Stink bug damage produced by the stylets in combination with saliva increased soybean cell wall thickness, possibly through lignin deposition, as shown by safranin-staining of attacked cotyledons. Moreover, NMR analysis of saliva identified small organic acids and amino acids, which may function as effectors of seed responses to stink bug damage. In addition, we used zymogram assays to measure activities of several hydrolytic enzymes such as, peroxidases, proteases, pectinases, amylases and a trehalase, suggesting an important digestive role of watery saliva. To our knowledge no previous study has reported metabolites, proteins, and digestive enzyme activity from *N. viridula’s* watery saliva saliva.

Similarities in feeding behavior among stink bug species by osmotic pumping of the seed content and plant fluids were used to explain the low variability that characterizes the anatomy of the mouthparts of these piercing-sucking insects^29,30^. In feeding, *N. viridula* uses a pair of external stylets to pierce and inject saliva produced by the salivary glands (Figs. 1, 2, 3). As described in other hemipterans^12,31^, these insects produce two types of saliva from their glands, jellified and watery. The jelling saliva forms a protuberance on the plant surface, called salivary sheath, to keep the stylets aligned as they lacerate and drill deep into the tissue (Fig. 3). Watery saliva disturbs the physiology and biochemistry of the tissues that surround the pierced canal. Maceration with the secretion and later withdrawal of the liquefied tissues lead to damaged zones, even further away from the inflicted wounds, collapsing the seed structure (Fig. 3, SI Fig. 1).

Although proteomic research on gut and salivary glands of *H. halys* has recently published^12,13,15^, there still remains a lack of knowledge on possible effectors of *N. viridula* watery saliva triggering the soybean defense responses^21,24,32^. To this end, we used NMR to investigate metabolites in the insect oral secretion, a powerful tool that has been barely used in entomology field^33^. Our NMR results showed that saliva of *N. viridula* was dominated by short-chain organic acids, like lactic acid and threo-isocitric acid, with acetic acid exhibiting the highest concentration (Fig. 4; Table 1). Threo-isocitric acid is a diastereomer of isocitrate and acts as the precursor of α-ketoglutarate in the Krebs cycle. We also identified several amino acids at high concentrations, like valine, glycine, leucine and glutamic acid (Fig. 4; Table 1). Isocitric acid is also a precursor to the amino acid biosynthesis of glutamine and glutamate. Glutamate may then be converted into γ-aminobutyric acid (GABA). In insects, GABA serves as a neurotransmitter, with a specific excitatory or inhibitory activity depending on the type of cell^34^. Conversely, although trehalose has been detected in haemolymph of seven species of aphids^35^, the NMR spectra of *N. viridula´s* saliva did not contain any sugar NMR peaks, including trehalose or glucose molecules (Fig. 4). We cannot rule out sugars at concentrations below our limits of detection, but a study on the velvet bean caterpillar, *Anticarsia gemmatalis* that measured sucrose consumption showed no sugars in the regurgitant, even after or during feeding process^36^.

Previous proteomic studies described the presence of the serine proteases trypsin and chymotrypsin in the saliva of *H. halys*^13^, suggesting some digestive activity. In this study, proteomic data and further validation with zymogram assays revealed that *N. viridula*’s watery secretion is mainly a fluid displaying digestive activity (Figs. 5, 6). We described a list of several enzymes, among them nine relevant hydrolases, several of which are related to carbohydrate hydrolysis, like amylases and a trehalase (Table 2). Enzymes related to the plant’s defense response, like metallo and serine peptidases, were also identified, most of them carrying secretion signal peptides (Table 2). Our proteomic analysis also revealed enzymes linked to oxidorectuctase activity, like malate dehydrogenase, peroxidase and cytochrome oxidases (Table 2). However, while the saliva tested positive for pectinase, amylase and proteolytic activities, no peroxidase activity was detected under native PAGE conditions (Fig. 6). Although peroxidases were not detected in watery saliva of *H. halys*, peroxidase activity was observed in the salivary sheath, and these proteins actually originated from the plants^12^. This suggests variability in the saliva activity between *N. viridula* and *H. halys*. Moreover, *N. viridula’*s saliva revealed the expression of the enzyme malate dehydrogenase in the oral secretion (Table 2). This is an enzyme that reversibly catalyses the oxidation of malate to oxaloacetate using the reduction of NAD+ to NADH. Taken together, our results show that *N. viridula*’s watery saliva is formed by different kinds of activated enzymes that may induce changes in damaged seeds.

Scarce information is available regarding the host targets of insect salivary effectors. Some evidence suggests that insect salivary proteins may be participating in signaling cascades through phosphorylation and/or dephosphorylation of plant proteins^20^. *N. viridula* herbivory phosphorylated and activated the mitogen-activated protein kinase (MAPK) pathway and induced an early peak of jasmonic and salicylic acid accumulation and ethylene emission in developing seeds of field-grown soybean; this up-regulated plant defenses and reduced stink bug preference^21^. Ethylene emission in soybean pods induced isoflavonoids, which are an effective defense against stink bugs^32,37^. Moreover, *N. viridula* feeding led to seed cell wall thickening as well as to increased expression of genes coding for expansine, xyloglucan endo-transferase, pectate lyase and polygalacturonase, all involved in the relaxation and restructuration of the cell wall^24^. Here we showed that in the presence of the insect saliva, but not with mechanical damage, seeds accumulated lignin in the lacerated area (Fig. 3). Furthermore, when attacked seeds reached maturity, they had a higher level of oxidative stress affecting the germinative potential (SI Fig. 1). These results strongly suggest that the watery saliva—not mechanical feeding activity—primarily induces direct plant defenses (e.g. walls strengthening by lignin accumulation). This, in turn, suggests that the oral secretion of *N. viridula* is somehow recognized by developing soybean seeds to induce tailored defenses against stink bugs.

The origin of biomolecules found in saliva has been the object of ongoing research, not only for stink bugs but also for other group of insects. The presence of symbiotic bacteria was reported in the gut tissue of the aphid *Acyrthosiphon pisum,* and removal of these bacteria reduced certain metabolites, including essential amino acids^38^. Similarly, the bacterial community colonizing the midgut of *N. viridula* could play a role in nutritional status and deactivation of soybean chemical defenses^39^. It has been proposed that stink bug saliva could carry yeasts and bacteria that eventually grow within the seeds^40^. Since we have previously depicted the salicylic acid accumulation in developing soybean seeds after *N. viridula* attack^21,24^, it is tempting to link this result to bacterial effectors present in the insect watery saliva. However, under laboratory conditions we were unable to obtain any bacterial growth from saliva samples (Virginia Medina personal communication). In addition, the axenic environment of *N. viridula* saliva was further supported by the analysis of our proteomic data against soybean, yeast and bacterial databases, which gave no positive hits. Similarly, the watery saliva study of *H. halys* did not produce evidence of any proteins from microorganisms^12^. Understanding the origin and mechanism of action of effectors produced by *N. viridula* will help to increase plant resistance against stink bugs.

Our study provides for the first time an insight on the composition of the watery saliva of *N. viridula*, an important pest for worldwide soybean crops, opening the door for more comprehensive analytical studies on the components of bug oral secretions as well as studies that link variabilities in the composition of stink bug saliva to different food sources.

## Material and methods

### Plant growth and treatments

Soybean seeds from the commercial variety Williams 82 (PI 518,671) were grown at the experimental fields of the University of Buenos Aires, Argentina. Experiments were carried out following regular agronomic practices and planting dates. Since the genetic variability in soybean is large, researchers have selected Williams 82 as variety to perform basic experiments. In order to test the effects of herbivory on soybean developing seeds, adults of *Nezara viridula* L. (Heteroptera: Pentatomidae) were collected from several rural locations near the city of Buenos Aires, and kept for 7 days under controlled conditions (25 ± 3 °C, 60 ± 9% relative humidity, and photoperiod of 15:9 L:D). After establishing a population of insects in the laboratory, young adults were used in the experiments. Stink bugs were starved for 24 h prior to performing the experiments, in order to enhance their feeding activity. For herbivory treatments, samples were collected after visual confirmation of stink bug feeding and damage produced by the stylet.

### Scanning electron microscopy (SEM) and histological analysis

Stink bug specimens were prepared for SEM by fixing with C_5_H_8_O_2_ (glutaraldehyde) using standard protocols and dried with a critical-point dryer (HCP-2, Hitachi Ltd., Tokyo, Japan). All dried samples were mounted on the metal stage of a SEM and coated with platinum particles by using an ion sputter (E101, Hitachi Ltd., Tokyo, Japan). Surface features of fresh or dry seeds with or without cuticles were also examined by SEM. Cuticles were removed from the seed coat surface by immersion in 1 M NaOH (sodium hydroxide) at 60 °C for 5 min. Then the samples were dehydrated by incubating in a series of EtOH (ethanol) 50–100% solutions and were completely dried. All samples were observed with a SEM (JSM-5310LV, JEOL Co., Tokyo, Japan) at 5 to 10 kV.

To evaluate salivary sheaths and wounds, treated pods were collected and stained to provide evidence of stink bug feeding. The staining solution was adopted from McBride’s^41^, and slightly modified, containing 0.2% acid fuchsin (Sigma) in 95% EtOH and CH_3_ COOH (glacial acetic acid) 1:1. Pods were submerged in this solution for 1 h and then rinsed in water. Dyed pods and cotyledons were then examined under a dissecting microscope.

For light microscopy analysis of pods and seeds, samples were processed as described previously^24^. Briefly, samples were fixed in FAA (33% formalin, 100% CH_3_ COOH and 70% EtOH, in a ratio of 5:5:90) and then embedded in paraffin. Cross sections of 10 µm were prepared with a microtome and mounted on slides. The deparaffinized sections were stained with safranin (Sigma), 1:25,000 in 50% EtOH for 30 min, and then with Fast Green (Sigma) 1:25,000 in 3:1 xylene: EtOH for 5 min during the dehydration process. The sections were cleared in xylene, mounted on a slide before observation with a Carl Zeiss light microscope.

### Watery saliva collection

To collect stink bug oral secretion, a group of 200 adults were chilled on ice to slow down their activity and metabolism, placed ventral side up and observed with a dissecting microscope. As the bugs returned to room temperature, the watery saliva was secreted from the tip of the beak. The saliva was collected by capillary with a pipet tip. For zymogram experiments, watery saliva was resuspended in PBS (Phosphate-Buffered Saline) pH 7.0. For proteomics, saliva was collected in 5 mM EDTA (Ethylenediaminetetraacetic acid) in 50 mM Tris–HCl pH 8.0 and stored at − 80 °C. Protein concentration was determined on a NanoDrop 2000 (Thermo Scientific, Wilmington, DE) and compared to a standard curve of BSA (bovine serum albumin).

### Metabolite analysis

Saliva samples were thawed and pooled in a final volume of 1.2 ml PBS and 2.4 ml of cold MeOH (methanol, HPLC-grade), following incubation at 20 °C for 20 min. After centrifuging the samples at 16,000 rcf for 30 min, protein pellets were discarded and the resulting supernatant was dried using a CentriVap Vacuum (Labconco, Kansas City, MO, USA). The concentrated samples were reconstituted in D_2_O (deuterium oxide), containing 1/9 mM of DSS-D6 (3-(Trimethylsilyl)-1-propanesulfonic acid-d6 sodium salt) as a reference and quantification standard, and vortexed until the pellets dissolved. The samples were then centrifuged at 14,000 rcf for 15 min at 4 °C before being transferred into 3 mm NMR tubes (Bruker Biospin, Billerica, MA, USA).

All experiments were run on a 600 MHz Avance III-HD (Bruker) NMR spectrometer equipped with a z-gradient triple resonance TCI cryoprobe and a Bruker SampleJet at the Complex Carbohydrate Research Center of the University of Georgia. 1D ^1^H and 2D ^1^H–^13^C HSQC and HSQC-TOCSY spectra were acquired at 25 °C. For the HSQC spectrum 32 scans were acquired with T1 and T2 acquisition times of 10 and 106 ms respectively and with the size of the FID being 512 and 2048 data points for F1 and F2. Similar parameters were employed for the acquisition of the HSQC-TOCSY spectrum. The resulting spectra were processed using Bruker Topspin 3.6 and MestreNova and metabolites were initially identified using a combination of AssureNMR (Bruker Biospin, USA, BBiorefcode metabolite database) and COLMARm^42^. The assignments were verified manually, and the metabolite identification is reported using a confidence level grade ranging from 1 to 5. A fairly long relaxation delay (d1 = 4 s) allowed us to approximately quantify identified metabolites by comparison with the DSS signal.

### Proteomics

To identify proteins in the watery saliva, sample digestion and Mass Spectrometry (MS) analysis were performed at CEQUIBIEM (https://cequibiem.qb.fcen.uba.ar/). Samples were reduced with 20 mM DTT (Dithiothreitol) for 45 min at 56 °C, alkylated with 55 mM C_2_H_4_INO (iodoacetamide) for 45 min in the dark and digested with trypsin (Promega V5111) overnight at 37 °C. NanoLC was carried out as previously described^43^. For data acquisition XCalibur 3.0.63 (Thermo Scientific) software was used. Q Exactive raw data were analyzed using Proteome Discoverer software (version 2.1.1.21 Thermo Scientific). Data were searched against specific databases for insects (taxid: 6960), soybean (taxid: 3847), bacteria (taxid:2), and yeasts (taxid: 147,537) (https://blast.ncbi.nlm.nih.gov). Only high confidence peptide matches with a maximum protein and peptide false discovery rate of 1% were selected through a reverse database approach.

### Zymograms for pectinolytic, amylolytic, peroxidase and proteolytic activities

An in-gel pectinase assay was performed using SDS-PAGE (Sodium dodecyl sulfate polyacrylamide gel electrophoresis) for visualizing pectinase activity. Saliva samples containing 25 µg of total protein were diluted in electrophoresis sample buffer (0.5 M Tris–HCl; pH 6.8, 20% glycerol, 2% SDS, 0.005% BPB-bromophenol blue) and loaded in a 10% gel. The substrate pectin (Sigma) was incorporated into the separating gel at final 1% concentration. After the run was finished, the gel was washed in citrate buffer plus 2.5% triton X-100 for 45 min and then incubated in citrate buffer for 60 min with gentle shaking. Finally, it was stained with 0.03% Ruthenium red until clear bands emerged.

For amylolytic activity testing the gels were co-polymerized with starch 0.5% as substrate. The electrophoretic run was performed in the adequate pH to the enzyme’s isoelectric point. After running, gels were immersed in a solution of 10 mM I_2_ (iodine) and 14 mM KI (potassium iodide) until the appearance of bands.

To visualize peroxidase activity, saliva samples were combined with native sample buffer (0.08 M Tris–HCl, pH 6.8, 30% glycerol and 0.02% BPB) and loaded onto 10% native gel in 1.5 M Tris–HCl, pH 8.8. The gels were then transferred to a peroxidase staining solution (2 mM dianisidine in 0.08 M phosphate buffer pH 7.0, 2% EtOH, 0.15% hydrogen peroxide) until the appearance of colored bands.

For proteolytic activity, native gels were copolymerized with 0.2% gelatin, and the samples were diluted in the specific buffer (0.4 M Tris–HCl, pH 6.8, 5% SDS, 20% glycerol and 0.03% BPB). After running, the gels were washed and incubated in developing buffer (5 mM CaCl_2_ in 50 mM Tris–HCl, pH 7.5) overnight at 37°C under gentle agitation. Gels were stained with 0.5% CBB (Coomassie Brilliant Blue, Thermo Fisher Scientific) in an aqueous solution of 40% MeOH and 10% CH_3_ COOH for 2 h. Gels were then distained in the same solution, without CBB, until the appearance of clear zones.

### Statistical analysis

All experiments, including proteomic analysis were performed with two biological replicates, each with three technical replicates. All analyses were performed with Prism 5.01 2007 (GraphPad Software Inc).

## Supplementary information

Supplementary information.

## References

[1] Molina GAR, Trumper EV (2012). Selection of soybean sods by the stink bugs, *Nezara viridula* and *Piezodorus guildinii*. J. Insect Sci..

[2] Panizzi AR (2015). Growing problems with stink bugs (Hemiptera: Heteroptera: Pentatomidae): Species invasive to the US and potential neotropical invaders. Am. Entomol..

[3] Rodrigues-Silva N, Canuto AF, Oliveira DF, Teixeira AF, Santos-Amaya OF, Picanço MC, Pereira EJ (2019). Negative cross-resistance between structurally different *Bacillus thuringiensis* toxins may favor resistance management of soybean looper in transgenic Bt cultivars. Sci. Rep..

[4] Peng Y, Wang K, Fu W, Sheng C, Han Z (2018). Biochemical comparison of dsRNA degrading nucleases in four different insects. Front. Physiol..

[5] Snodgrass GL, Adamczyk JJ, Gore J (2005). Toxicity of insecticides in a glass-vial bioassay to adult brown, green, and southern green stink bugs (Heteroptera: Pentatomidae). J. Econ. Entomol..

[6] Lee DH, Wright SE, Leskey TC (2013). Impact of insecticide residue exposure on the invasive pest, *Halyomorpha halys* (Hemiptera: Pentatomidae): Analysis of adult mobility. J. Econ. Entomol..

[7] Olson D, Ruberson J, Zeilinger A, Andow D (2011). Colonization preference of *Euschistus servus* and *Nezara viridula* in transgenic cotton varieties, peanut, and soybean. Entomol. Exp. Appl..

[8] Panizzi AR (1997). Wild hosts of pentatomids: Ecological significance and role in their pest status on crops. Annu. Rev. Entomol..

[9] Tood JW, Turnipseed SG (1974). Effects of southern green stink bug damage on yield and quality of soybeans. J. Econ. Entomol..

[10] Felton GW, Chung SH, Hernandez MGE, Louis J, Peiffer M, Tian D, Voelckel C, Jander G (2014). Herbivore oral secretions are the first line of protection against plant-induced defences. Annual Plant Reviews, Insect–Plant Interactions.

[11] Boulain H, Legeai F, Guy E, Morliere S, Douglas NE, Oh J, Murugan M, Smith M, Jaquiéry J, Peccoud J, White FF, Carolan JC, Simon JC, Sugi A (2018). Fast evolution and lineage-specific gene family expansions of aphid salivary effectors driven by interactions with host-plants. Genom. Biol. Evol..

[12] Peiffer M, Felton GW (2014). Insights into the saliva of the brown marmorated stink bug *Halyomorpha halys* (Hemiptera: Pentatomidae). PLoS ONE.

[13] Lomate PR, Bonning BC (2016). Distinct properties of proteases and nucleases in the gut, salivary gland and saliva of southern green stink bug,* Nezara viridula*. Sci. Rep..

[14] Lomate PR, Bonning BC (2018). Proteases and nucleases involved in the biphasic digestion process of the brown marmorated stink bug, *Halyomorpha halys* (Hemiptera: Pentatomidae). Arch. Insect Biochem. Physiol..

[15] Serteyn L, Francis F (2019). Insight into salivary gland proteomes of two polyphagous stink bugs: *Nezara viridula* L. and *Halyomorpha halys* Stål.. Proteomics.

[16] Cristofoletti PT, Ribeiro AF, Terra WR (2005). The cathepsin L-like proteinases from the midgut of *Tenebrio molitor* larvae: Sequence, properties, immunocytochemical localization and function. Insect Biochem. Mol. Biol..

[17] Hopke J, Donath J, Blechert S, Boland W (1994). Herbivore-induced volatiles—The emission of acyclic homoterpenes from leaves of *Phaseolus lunatus* and *Zea mays* can be triggered by a beta-glucosidase and jasmonic acid. FEBS Lett..

[18] Mattiacci L, Dicke M, Posthumus MA (1995). β-Glucosidase: An elicitor of herbivore-induced plant odor that attracts host-searching parasitic wasps. Proc. Natl. Acad. Sci. USA.

[19] Schmelz EA, Carroll MJ, LeClere S, Phipps SM, Meredith J, Chourey PS, Alborn HT, Teal PEA (2006). Fragments of ATP synthase mediate plant perception of insect attack. Proc. Natl. Acad. Sci. USA.

[20] Bonaventure G, Voelckel C, Jander G (2014). Plants recognize herbivorous insects by complex signaling networks. Plant Insect Interactions.

[21] Giacometti R, Barneto J, Barriga LG, Sardoy PM, Balestrasse K, Andrade AM, Pagano EA, Alemano SG, Zavala JA (2016). Early perception of stink bug damage in developing seeds of field-grown soybean induces chemical defences and reduces bug attack. Pest Manag. Sci..

[22] Liu Y, Wang WL, Guo GX, Ji XL (2009). Volatile emission in wheat and parasitism by *Aphidius avenae* after exogenous application of salivary enzymes of *Sitobion avenae*. Entomol. Exp. Appl..

[23] Ma R, Chen JL, Cheng DF, Sun JR (2010). Activation of defense mechanism in wheat by polyphenol oxidase from aphid saliva. J. Agric. Food Chem..

[24] Giacometti R, Ilina N, Eduardo PA, Zavala JA (2018). Stink bug *Nezara viridula* sustains late MAPKs phosphorylation status and induces expression of genes related with cell wall rearrangement in developing soybean seeds. Arthropod-Plant Interact..

[25] Hogenhout SA, Bos JI (2011). Effector proteins that modulate plant–insect interactions. Curr. Opin. Plant Biol..

[26] Turlings TC, Alborn HT, Loughrin JH, Tumlinson JH (2000). Volicitin, an elicitor of maize volatiles in oral secretion of *Spodoptera exigua*: Isolation and bioactivity. J. Chem. Ecol..

[27] Halitschke R, Schittko U, Pohnert G, Boland W, Baldwin IT (2001). Molecular interactions between the specialist herbivore Manduca sexta (Lepidoptera, Sphingidae) and its natural host *Nicotiana attenuata*. III. Fatty acid-amino acid conjugates in herbivore oral secretions are necessary and sufficient for herbivore-specific plant responses. Plant Physiol..

[28] Yoshinaga N, Morigaki N, Matsuda F, Nishida R, Mori N (2005). In vitro biosynthesis of volicitin in *Spodotera litura*. Insect Biochem. Mol. Biol..

[29] Depieri RA, Panizzi AR (2011). Duration of feeding and superficial and in-depth damage to soybean seed by selected species of stink bugs (Heteroptera: Pentatomidae). Neotrop. Entomol..

[30] Silva FAC, da Silva JJ, Depieri RA, Panizzi AR (2012). Feeding activity, salivary amylase activity, and superficial damage to soybean seed by adult *Edessa meditabunda* (F.) and *Euschistus heros* (F.) (Hemiptera: Pentatomidae). Neotrop. Entomol..

[31] Miles PW (1968). Insect secretions in plants. Annu. Rev. Phytopathol..

[32] Zavala JA, Mazza CA, Dillon FM, Chludil HD, Ballare CL (2015). Soybean resistance to stink bugs (*Nezara viridula* and *Piezodorus guildinii*) increases with exposure to solar UV-B radiation and correlates with isoflavonoid content in pods under field conditions. Plant Cell Environ..

[33] Snart CJ, Hardy IC, Barrett DA (2015). Entometabolomics: Applications of modern analytical techniques to insect studies. Entomol. Exp. Appl..

[34] Gisselmann G, Plonka J, Pusch H, Hatt H (2004). *Drosophila melanogaster* GRD and LCCH3 subunits form heteromultimeric GABA-gated cation channels. Br. J. Pharmacol..

[35] Moriwaki N, Matsushita K, Nishina M, Kono Y (2003). High concentrations of trehalose in aphid hemolymph. Appl. Entomol. Zool..

[36] Wei X, Johnson SJ, Hammond AM (1998). Sugar-feeding strategy of adult velvetbean caterpillar (Lepidoptera: Noctuidae). Environ. Entomol..

[37] Dillon FM, Tejedor MD, Ilina N, Chludil HD, Mithöfer A, Pagano EA, Zavala JA (2018). Solar UV-B radiation and ethylene play a key role in modulating effective defenses against *Anticarsia gemmatalis* larvae in field-grown soybean. Plant Cell Environ..

[38] Wang Y, Carolan JC, Hao F, Nicholson JK, Wilkinson TL, Douglas AE (2010). Integrated metabonomic–proteomic analysis of an insect-bacterial symbiotic system. J. Proteome Res..

[39] Medina V, Sardoy PM, Soria M, Vay CA, Gutkind GO, Zavala JA (2018). Characterized non-transient microbiota from stinkbug (*Nezara viridula*) midgut deactivates soybean chemical defenses. PLoS ONE.

[40] Clarke RG, Wilde GE (1970). Association of the green stink bug and the yeast-spot disease organism of soybeans. 1. Length of retention, effect of molting, isolation from feces and saliva. J. Econ. Entomol..

[41] McBride MC (1936). A method of demonstrating rust hyphae and haustoria in unsectioned leaf tissue. Am. J. Bot..

[42] Bingol K, Li DW, Zhang B, Brüschweiler R (2016). Comprehensive metabolite identification strategy using multiple two-dimensional NMR spectra of a complex mixture implemented in the COLMARm web server. Anal. Chem..

[43] Piccinni FE, Ontañon OM, Ghio S, Sauka DH, Talia PM, Rivarola ML, Valacco MP, Campos E (2019). Secretome profile of *Cellulomonas* sp. B6 growing on lignocellulosic substrates. J. Appl. Microbiol..

