# Supplementary material for: Digestive activity and organic compounds of Nezara viridula watery saliva induce defensive soybean seed responses
Source: Sci Rep. 2020 Sep 22;10:15468. doi: 10.1038/s41598-020-72540-3 (PMC7508886; doi:10.1038/s41598-020-72540-3)

**Digestive activity and organic compounds of *Nezaraviridula* watery saliva induce defensive soybean seed responses**

Romina Giacometti^1-2^, Vanesa Jacobi^1^, Florencia Kronberg^1-2^, Charalampos Panagos^3^ , Arthur S. Edison^3^ and Jorge A. Zavala^1-2*^

^1^ CONICET-Consejo Nacional de Investigaciones Científicas / Instituto de Investigaciones en Biociencias Agrícolas y Ambientales, Facultad de Agronomía, Universidad de Buenos Aires, Avda. San Martín 4453, C1417DSE Buenos Aires, Argentina.

^2^Universidad de Buenos Aires, Facultad de Agronomía, Cátedra de BioquímicaAvda. San Martín 4453, C1417DSE Buenos Aires, Argentina.

^3^Complex Carbohydrate Research Center (CCRC), University of Georgia, Athens, GA, United States

* Correspondence: [zavala@agro.uba.ar](mailto:zavala@agro.uba.ar)

Tel: +54-11-5287-0530. FAX: +54-11-4-524-8087

**SUPPLEMENTARY INFORMATION**

Additional supporting information may be found in the online version of this article:

**Seed damage and germinative analysis**

Mature treated and control soybean seeds without cuticles were used for SEM analysis. In the laboratory, 80 mature seeds (R8) that were previously exposed to stink bugs feeding in enclosed mesh bags for 24 or 48 h period, and 80 control seeds from non-treated plants were sterilized and soaked in deionized (DI) water for 30 min to soften seeds coats, and kept in a germination chamber (25 ± 1 °C) for 5 days in absence of light. Seeds with coleoptiles longer than 2 mm were scored as positive for the test.

**Peroxidase activity**

For native PAGE, 10 µg of protein from soybean seeds extracts were combined with native sample buffer (0.08 M Tris-HCl, pH 6.8, 30% glycerol and 0.02% BPB) and loaded onto a 10% acrylamide gel in 1.5 M Tris-HCl, pH 8.8 and electrophoresed at 100 V for 4 h. The gel was then transferred to a peroxidase staining solution (2 mM dianisidine in 0.08 M PBS pH 7.0, 2% EtOH, 0.15% hydrogen peroxide) to visualize activity.


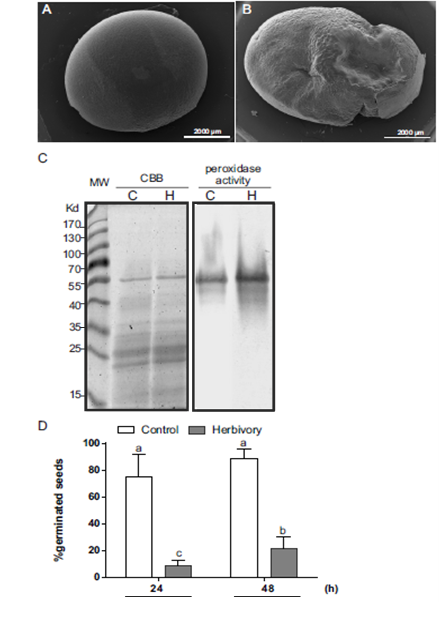


**Supplementary Figure 1. Effect of *Nezara* feeding on mature soybean seeds.** SEM analysis of mature soybean cotyledons after removal of the tegument: **A.** control seed, and **B.** attacked by the insect. **C.**The gel on the right shows a zymogram analysis to detect soybean seed´s peroxidase activity in control (C) and attacked seeds (H). The gel on the left shows a CBB image that corresponds to the coomasie brilliant blue staining of an independent gel as loading control, one of three independent experiments is shown. Aprestained molecular weight marker was used (MM, Kaleidoscope, Bio-Rad). The white space shows the separation of two independent gels.**D.** Soybean seeds germinative power. Control seeds and seeds exposed to 24 h or 48 h herbivory treatment were harvested and germinated for 5 days in the dark under controlled environmental conditions. Results are shown as an average of the score of 80 seeds used per treatment, bars indicate SD, different letters indicate significant differences (p < 0.05) according to Tukey's multiple range test.

**Supplementary Figure 2. Original gels in Supplementary Figure 1. A.**Loading control gel stained with CBB and**B.** zymogram analysis for peroxidase activity in control seeds.

**A B**

**
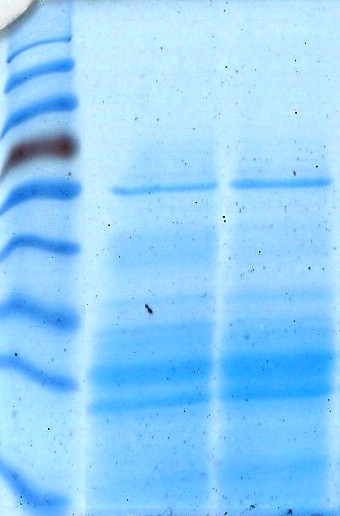
**
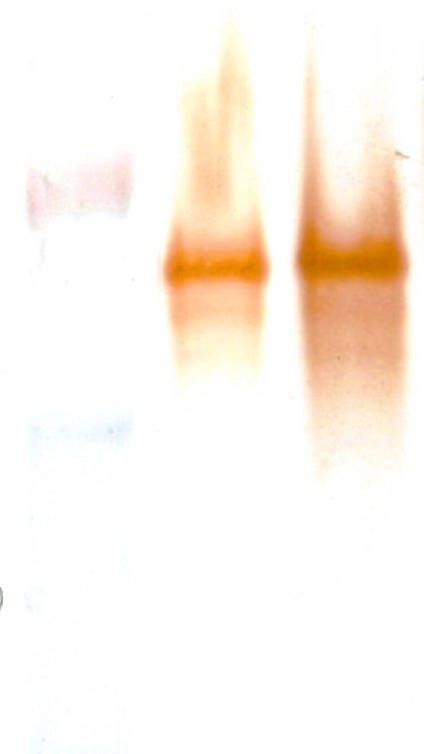


**Supplementary Figure 3. Original gels in Figure 6. A.** Zymogram for pectinolytic activity. **B.** amylase activity.**C.** peroxidase activity. **D.** protease activity.

**A B**


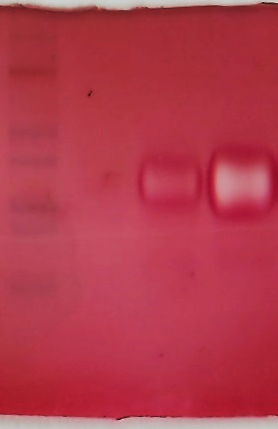

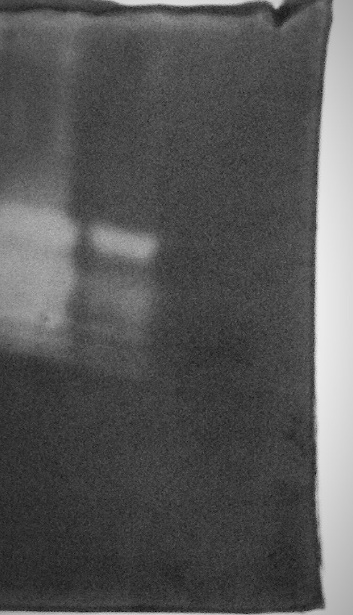


**C D**


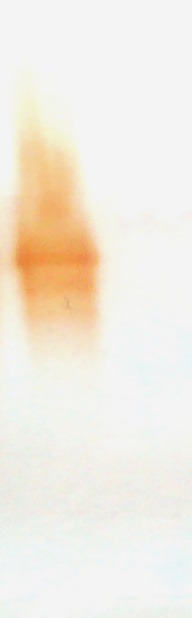

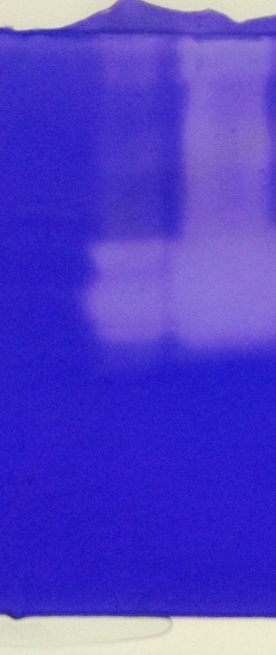

Supplement: Supplementary file 1 — Supplementary information. [file 41598_2020_72540_MOESM1_ESM.docx]
